# CovR-Controlled Global Regulation of Gene Expression in *Streptococcus mutans*

**DOI:** 10.1371/journal.pone.0020127

**Published:** 2011-05-31

**Authors:** Alexander Dmitriev, Saswat S. Mohapatra, Patrick Chong, Melody Neely, Saswati Biswas, Indranil Biswas

**Affiliations:** 1 Department of Microbiology, Molecular Genetics and Immunology, University of Kansas Medical Center, Kansas City, Kansas, United States of America; 2 Department of Molecular Microbiology, Institute of Experimental Medicine, Saint-Petersburg, Russia; 3 Department of Microbiology and Immunology, Wayne State School of Medicine, Detroit, Michigan, United States of America; East Carolina University, United States of America

## Abstract

CovR/S is a two-component signal transduction system (TCS) that controls the expression of various virulence related genes in many streptococci. However, in the dental pathogen *Streptococcus mutans*, the response regulator CovR appears to be an orphan since the cognate sensor kinase CovS is absent. In this study, we explored the global transcriptional regulation by CovR in *S. mutans*. Comparison of the transcriptome profiles of the wild-type strain UA159 with its isogenic *covR* deleted strain IBS10 indicated that at least 128 genes (∼6.5% of the genome) were differentially regulated. Among these genes, 69 were down regulated, while 59 were up regulated in the IBS10 strain. The *S. mutans* CovR regulon included competence genes, virulence related genes, and genes encoded within two genomic islands (GI). Genes encoded by the GI TnSmu2 were found to be dramatically reduced in IBS10, while genes encoded by the GI TnSmu1 were up regulated in the mutant. The microarray data were further confirmed by real-time RT-PCR analyses. Furthermore, direct regulation of some of the differentially expressed genes was demonstrated by electrophoretic mobility shift assays using purified CovR protein. A proteomic study was also carried out that showed a general perturbation of protein expression in the mutant strain. Our results indicate that CovR truly plays a significant role in the regulation of several virulence related traits in this pathogenic streptococcus.

## Introduction

*Streptococcus mutans*, a gram-positive bacterium that resides in the human oral cavity, is considered to be the primary causative agent of dental caries [1], [2]. *S. mutans* has developed several unique mechanisms that allows for the successful survival, colonization, and continual presence in the oral cavity. *S. mutans* uses the dietary carbohydrates of its host to produce an extracellular sticky polysaccharide known as glucan, which is essential for anchoring to the tooth surface, forming biofilms, commonly known as dental plaque [3]. *S. mutans* also produces lactic acid as a byproduct from the metabolism of carbohydrates ingested by its host [4]. In the dental plaque, where the pH can be as low as 3.0 after exposure to carbohydrates [5], *S. mutans* induces an acid tolerance response that allows this pathogen to survive and grow under conditions of low-pH [6]. The localized drop in pH also leads to demineralization of the tooth enamel, promoting the formation of dental caries. Oral bacteria, including *S. mutans*, can also enter the blood stream during dental procedures, and cause transient bacteremia and infective endocarditis [1]; some reports suggest that as much as 14% of viridians streptococcus-induced endocarditis cases may be linked to *S. mutans* [2], [7]. The extraordinary ability of *S. mutans* to adapt and persist in the human oral cavity is due to its ability to rapidly respond and adapt to the ever changing conditions of the oral cavity, including changes in the availability of essential nutrients, fluctuations in oxidative and osmotic stress conditions, and variations of temperature and pH.

Two-component signal transduction systems (TCS) are the predominant mechanisms by which bacteria sense changes in their external or internal environment [8]. TCSs are involved in the regulation of gene expression in response to various environmental cues. Although several different kinds of TCS exist, the fundamental model of a TCS consists of a sensor kinase that is usually located at the cell surface or periplasmic space, facilitating the rapid detection of external signals (for recent reviews, see [8], [9], [10], [11], [12]. Detection of an appropriate signal leads to a conformational change, which results in autophosphorylation of the protein. Typically, a conserved histidine residue in the sensor kinase receives a phosphoryl group from ATP, followed by transfer of the phosphoryl group from the kinase to the cognate response regulator. The response regulator is composed of two functional components: a receiver domain with a conserved phosphorylatable aspartic acid residue, and an effector domain that is activated upon phosphorylation of the aspartate residue. Phosphorylation of the response regulator alters its ability to interact with either the target DNA sequence, or the RNA polymerase, in order to activate or repress transcription of one or more target genes in response to the signal received by the sensor kinase. Coordinated gene expression in response to environmental signals is particularly important for many human pathogens [13], [14].

*S. mutans* encodes at least 14 TCS that play important roles in bacterial adaptation, bacteriocin production, and biofilm formation [15], [16]. Of these, CovR/S is one of the most important and widely studied TCSs in *S. mutans* [17], [18]. In the case of group A streptococcus (GAS), the bacterium in which the CovR/S system was first characterized [19], the TCS regulates about 15% of the genes, either directly or indirectly [20], [21]. These include the *has* operon (hyaluronic acid capsule synthesis), *ska* (streptokinase), *sagA* (streptolysin S), and *speB* (cysteine protease B) [19], [22], [23]. CovR is also required for the expression of virulence related genes in group B- (GBS) and C-streptococcus (GCS) [21], [24], [25]. As much as 6% of the genes of GBS are regulated by CovR/S, including cytolysin and CAMP factor, two key virulence determinants [24]. In both GAS and GBS, CovR regulates the expression of common sets of genes in different strains; however, the repertoire of genes regulated by CovR may also vary depending on the particular strain [21], [26], [27]. Unlike most response regulators, in GAS and GBS CovR predominantly acts as a repressor of most of the genes that it regulates, including its own expression [24], [28], [29], [30], [31]. Regulation of gene expression by CovR may be indirect, involving another regulator [32], or direct, by binding to the promoter region of the target genes [24], [28], [29], [30], [31]. In *S. mutans*, CovR has also been shown to repress the expression of various virulence factors, including *gtfB/C* (glucosyltransferase B/C), *gbpC* (glucan-binding protein C), and also autoregulates its own expression; CovR directly regulates these genes by binding to the promoter regions [17], [18], [33].

Transcriptomicanalysis in GAS and GBS indicates that although CovR acts as a transcriptional repressor, the expression of some genes are down regulated in the isogenic *covR* mutant strains [21], [24], [27], [32]. However, direct binding by CovR to the target promoter was only shown for the promoter of the *dppA* gene [32], and the *cfb* gene [27], both of which are up-regulated genes identified by microarray analysis. Moreover, an *in vitro* transcription assay indicates that in addition to CovR, other cellular factors are necessary for activation of the *dppA* promoter [32]. We have recently found that *S. mutans* CovR also activates expression of the SMU.1882 gene. As with GAS *dppA* expression, activation also requires additional cellular factors [34].

Multiple mechanisms have been proposed for the modulation of CovR activity [35]. Although it is thought that the cognate sensor kinase, CovS, phosphorylates the conserved aspartate residue on the CovR for its activation, it has never been demonstrated *in vivo* or *in vitro*. In contrast, it is proposed that CovS dephosphorylates CovR under certain stress conditions, thereby inactivating its repressor function [36]. In GBS, a eukaryotic-like serine-threonine kinase, STK, was also shown to modulate CovR activity by phosphorylating a threonine residue on the N-terminal receiver domain. However, this modulation may be specific for GBS CovR, and was not shown for GAS CovR. In the case of *S. mutans*, CovR appears to be an orphan response regulator, since no CovS was found in this streptococcus, and a consensus binding sequence (CBS) for CovR has not been clearly identified. The goal of the present study was to analyze CovR-controlled regulation in *S. mutans* strain UA159 at the transcriptional level, and their manifestations at the translational and phenotypic levels.

## Materials and Methods

### Bacterial strains and growth conditions

*S. mutans* strain UA159 and its isogenic *covR*-deleted mutant strain IBS10 (Table S1) were grown in Todd-Hewitt medium (BBL; Becton Dickinson) supplemented with 0.2% yeast extract (THY). The pH of the THY medium was routinely adjusted with HCl to 7.2 prior to sterilization. For growth kinetic analysis, 2 ml of overnight cultures were inoculated in 38 ml of the THY broth, and grown anaerobically in Klett flasks at 37°C. The optical densities of the cultures were monitored by using a Klett-Summerson colorimeter [37] with a red filter, or measured at OD_600_ using a spectrophotometer (Bio-Rad Laboratories, USA). When necessary, erythromycin (Em, 5 µg/ml), kanamycin (Km, 300 µg/ml), or spectinomycin (Sp, 300 µg/ml) was added to liquid or solid growth medium.

### Construction of *covR* deleted strains

Two different *covR* deleted strains, IBS06 and IBS10, were used in this study, and the construction of the latter strain, a derivative of UA159, was previously described [18]. Similar to the IBS10 strain, IBS06 is a derivative of NG-8, in which the *covR* gene was disrupted by gene-replacement. Briefly, plasmid pIB10 [18], which contains a 1.7-kb DNA fragment containing the entire *covR* gene cloned into the pGEM-T-Easy vector (Promega), was digested by *Mfe*I at a unique site within the *covR* coding sequence. A kanamycin resistance cassette (ΩKm), isolated from plasmid pUC4ΩKm [38] after digestion by *Eco*RI, was ligated into the *Mfe*I-digested site, and the resulting construct was named pIB11. The orientation of the kanamycin resistance cassette was verified by PCR. Plasmid pIB11 was linearized by *Not*I, and then used for the transformation of NG-8 strain [39]. Transformants were selected on THY agar containing kanamycin, and designated as IBS06 (NG-8). PCR analysis with flanking primers and Southern hybridization using the entire *covR* gene as probe against IBS06 chromosomal DNA as template was performed to confirm that *covR* inactivation had occurred in IBS06 by double-crossover recombination.

### Transformation of *S. mutans*

Transformation of *S. mutans* was done as previously described [40]. Briefly, 0.5 ml of the overnight culture and 0.5 ml of heat inactivated horse serum were inoculated in 10 ml of THY broth, and cultured to OD_600_ = 0.2. At this point, competence stimulating peptide was added to the final concentration of 500 ng/ml. One ml of the medium was transferred into a fresh tube, and 200 ng of DNA was added. The strains were incubated at 37°C for 90 min, and transformants were selected on THY-agar in the presence of appropriate antibiotics.

### Semiquantitative RT- PCR (sqRT-PCR)

RNA samples were isolated from the *S. mutans* cultures grown to mid-exponential growth phase (70 Klett units) following a previously described protocol [33]. RNA samples were quantified using a UV spectrophotometer. The sqRT-PCR analyses were performed using a two tube RT PCR system. One microgram of RNA was used for first strand cDNA synthesis (at 42°C, one hr incubation) using Superscript-II reverse transcriptase (Invitrogen, CA). The reaction was terminated by incubating the reaction tubes at 70°C for 15 min, followed by RNaseH (Invitrogen, CA) treatment at 37°C for 20 min, and purification of the cDNA using a PCR purification column (Qiagen). The concentration of cDNA was determined using a UV spectrophotometer. Five nanograms of cDNA were used as template for PCR amplification using gene specific primers as listed in Table S1. Products obtained from the sqRT- PCR reactions were loaded onto a 1% agarose gel, photographed, and quantified using Doc-It-LS (UVP) software. The expression of *gyrA* serves as the internal control to ensure that equal amounts of total RNA were used in each sqRT-PCR reaction.

### Real time RT-PCR

Quantitative real-time PCR was performed as previously described [41]. Briefly, the same RNA samples that were used for sqRT-PCR were subjected to one-tube quantitative SYBR green PCR assay using a Power SYBR Green RNA kit (Applied Biosystems), and employing an ABI-Prism 7000 LightCycle system (Applied Biosystems). The primers used for real-time PCR were the same as the sqRT-PCR primers. As an additional control for each primer set and RNA sample, the cDNA synthesis reaction was carried out in the absence of reverse transcriptase to verify that genomic DNA did not contaminate the RNA samples. The critical threshold cycle (C_t_) was defined as the cycle in which fluorescence was detectable above the background, and is inversely proportional to the logarithm of the initial number of RNA molecules. A standard curve was plotted for each primer set with C_t_ values obtained from amplification of known quantities of DNA. The standard curve was used for transformation of the C_t_ values to the relative number of cDNA molecules. The expression levels of all the genes tested by real time RT-PCR were normalized using the *gyrA* expression as an internal standard. Each RT-PCR was performed with at least two-independent RNA samples in duplicate, and the x-fold change of the transcription level was calculated with the ABI Prism SDS Software. Student's t-test was used to calculate the significance of the difference between the mean expression of a given gene in the wild-type and its mean expression in the *covR* deleted strain.

### DNA microarray analysis

Affymetrix NimbleExpress arrays were purchased from Affymetrix (Santa Clara, CA). The arrays were designed based on the *S. mutans* strain UA159 genome sequence [15], and are MIAME compliant. The arrays were represented by 5 blocks, and each block consisted of 1960 predicted *S. mutans* ORFs. cDNA synthesis, biotinylation, and DNA microarray hybridization was done as recommended by the manufacturer. For both the strains (UA159 and IBS10), two independently isolated RNA samples were analyzed. The change of gene expression levels (*n*-fold) between UA159 and IBS10 strains was calculated by taking the ratio of corresponding average signal intensities with ArrayStar software, version 3.0 (DNASTAR, Inc, Madison, WI). Student's *t*-test was applied, and the genes with 1.5-fold difference in RNA levels (*P*-values≤0.05) were considered to be differentially expressed. The microarray experiments were done according to MIAME standard, and all of the microarray data are available through the Gene Expression Omnibus data repository at NCBI (www.ncbi.nlm.nih.gov/geo/) via accession number GSE16500.

### Electrophoretic mobility shift assay (EMSA)

CovR protein was previously expressed in *E. coli* and purified as described [34]. After quantification by Bradford reagent using BSA as standard, the purified CovR was used for EMSA analysis, as described before [34]. Briefly, target promoters were PCR amplified, purified, and radio-labeled using T4 polynucleotide kinase (T4 PNK, New England Biolabs) as previously described [18]. The labeled PCR fragment (0.1 pmole) was incubated with increasing concentrations of CovR in a binding buffer that contains 50 mM NaPO_4_ (pH 6.5), 50 mM NaCl, 1 mM MgCl_2_, 1 mM CaCl_2_, 1 mM dithiothreitol, 2 µg/ml poly (dI-dC), and 10% glycerol. Each binding reaction was performed in a 40 µl volume, and incubated at room temperature for 45 min. After incubation, the samples were loaded onto a 4.0% native acrylamide gel containing 50 mM NaPO_4_ buffer (pH 6.5) and 4% glycerol. Following electrophoresis, the gel was dried and analyzed by a Typhoon scanner following exposure of the dried gel to a phosphor imager screen.

### Analysis of total cellular lysate

Cultures were grown in THY, collected by centrifugation at the indicated growth phases, and washed twice in a half volume of phosphate buffered saline (PBS). Total cell extracts were prepared by lysing the cell suspension with a bead beater (MP Biomedicals) as previously described [42]. Total crude lysates (50 µg) were loaded on to a 4–20% gradient SDS-PAGE, and stained with PageBlue (Fermentas). The band of interest was excised from the stained gel and subjected to mass-spectrometry analysis.

### Two-dimensional gel electrophoresis (2-DE)

*S. mutans* cultures were grown until the mid-exponential phase corresponding to ∼70 Klett units. The cells were collected by centrifugation, and suspended in 500 µl of osmotic lysis buffer (10 mM Tris-HCl, pH 7.4, 0.3% SDS) containing protease inhibitors. Proteins were isolated using FastPrep protein isolation and PlusOne 2-D Clean-up kits (GE Healthcare, Piscataway, NJ, USA) to remove non-protein contaminants. Before loading, the samples were diluted to 1.0 mg/ml in SDS-boiling buffer (60 mM Tris-HCl, 5% SDS, 10% glycerol) and placed in boiling water bath for 5 min. A total of 50 ug of each protein sample was subjected to isoelectric focusing (IEF), which was carried out in glass tubes of inner diameter 2.0 mm using 2% pH 4–8 ampholines. Fifty nanograms of IEF internal standard, tropomyosin, was included in each samples. This protein migrates as a doublet with lower polypeptide spot of MW 33 kD and pI5.2; an arrow on the stained gel marks its position. After equilibration for 10 min in buffer “0” (10% glycerol, 50 mM dithiothreitol, 2.3% SDS, 0.0625 M Tris-HCl, pH 6.8) each tube gel was sealed to the top of a stacking gel, which was on the top of a 10% acrylamide slab gel (0.75 mm thick). SDS-PAGE was carried out for about 4 hrs at 15 mA/gel. Digital images of silver stained gels were acquired with a Typhoon 9410 imager (GE Healthcare). The whole cell lysates of both UA159 and IBS10 strains were prepared from two independently grown cultures, and the representative images are shown. Analysis of the gels, including protein spot detection and quantitation, was done with PDQuest software (Bio-Rad Laboratories). Gels were normalized based on the sum of all protein spots detected in each sample.

### Protein spot identification

The proteins of interest were excised from the SDS-PAGE gels with a robotic spot cutter (Bio-Rad Laboratories), and identified with tandem mass-spectrometry, as previously described [43]. The spectra were obtained, and the MASCOT software (www.matrixscience.com) was used to analyze them against NCBI *S. mutans* specific database.

## Results

### CovR-regulon of *S. mutans* strain UA159

To investigate the function of CovR in *S. mutans* strain UA159, we used a previously constructed Δ*covR* mutant strain, IBS10 [18]. In this strain, a non-polar *aad9* gene conferring spectinomycin resistance has been inserted into *covR*. To explore the global regulatory role of CovR in *S. mutans*, we performed a whole-genome transcriptome analysis by comparing the gene expression pattern of the exponentially grown (70-Klett unit) cultures of IBS10 and UA159 using a high-density NimbleGen microarray chip. Exponentially grown cultures were chosen because *covR* transcription appears to be optimum at this growth phase [17]. ArrayStar software (DNASTAR, Inc) was used, and 1.5-fold cuftoff in gene expression was applied (*P*-values≤0.05) to identify the genes differently transcribed and therefore affected by CovR. Transcriptome analysis revealed that the transcription of 69 genes was down regulated, while the transcription of 59 genes was up regulated in the IBS10 strain. Several differentially expressed genes could be clustered into many groups (Table S2), such as genes associated with virulence, competence, house keeping functions, and genes associated with genomic islands (GI).

We found several virulence-associated genes differentially expressed in IBS10 (Table S2). Genes such as *gtfB*, *gtfC*, and *gbpC* were previously shown to be repressed by CovR in *S. mutans* [17], [18], [33]. In addition to these three genes, our transcriptome analysis found that three additional genes, including *wapE* and *ftf*, were also up regulated in IBS10 (Table S2). On the other hand, two virulence-associated genes were found to be positively regulated by CovR: *spaP*, whichencodes a well-studied surface antigen AgI/II [44], and *patB*, which putatively encodes a hemolysin (Table S2).

The *S. mutans* genome contains multiple GIs [15], which are acquired by the organism through horizontal gene transfer. Among these GIs, the presence of TnSmu1 and TnSmu2 among the different isolates had previously been studied [45], [46]. TnSmu1, which lies adjacent to a cluster of tRNA genes, corresponds to a large region of 23 kb spanning from SMU.191 to SMU.226; this region encodes many predicted transposases, integrases, transporter proteins, and hypothetical proteins. At least 11 genes were induced in the strain without functional *covR* compared to the wild-type strain, UA159.

In contrast, several genes within TnSmu2 were down regulated in the Δ*covR* mutant IBS10. TnSmu2 is the largest genomic island (57 kb) found in the *S. mutans* genome, and it contains about 47 genes organized into several operons [15]. The largest operon within this genomic island is the *smt* operon [47], which is approximately 32-kb in length. The *smt* operon includes 10 genes with high homology degree of similarity to several secondary-metabolite biosynthesis genes. Expression of all 10 of the genes in the *smt* operon (SMU.1339 to SMU.1348) was dramatically decreased in the *covR* mutant strain (Table S2). In fact, the fold difference in the expression of these genes was the highest of all the genes that were differentially regulated in the mutant. TnSmu2 also contains two genes, SMU.1365 and SMU.1366, which are generated due to gene duplication of SMU.1347 and SMU.1348, respectively [45]. Expression of SMU.1365 and SMU.1366 was also reduced (7.6- and 8.0-fold, respectively) in the mutant strain IBS10. A putative transcriptional regulator of the TetR/AcrR family, SMU.1349, is present just upstream of the *smt* operon, and is transcribed divergently. In contrast to the *smt* operon genes, expression of SMU.1349 was not significantly altered in the Δ*covR* mutant strain (data not shown).

The expression of another gene in the genomic island GI-4 was also differentially regulated in the Δ*covR* mutant strain IBS10. GI-4 is a small genomic island less than 10 kb in length, and includes about 12 genes, two of which encode a sorbose phosphotransferase (PTS) gene cluster (SMU.100 and SMU.101). The expression of SMU.100 was down regulated in IBS10. Thus, taken together, it appears that the expression levels of several horizontally transferred genes were differentially affected by the *covR* inactivation.

We also observed that the expression levels of at least four oxidative stress-related genes were differentially regulated in the Δ*covR* mutant (Table S2). However, the fold differences in the expression levels between the mutant and the wild-type strains were modest; the greatest difference was 2.4-fold for the SMU.758 gene that encodes a putative NADH dehydrogenase. Thus, the results suggest that CovR probably plays an important role in oxidative stress response in *S. mutans*.

### Confirmation of differential gene expression by quantitative PCR

**Figure 1:**
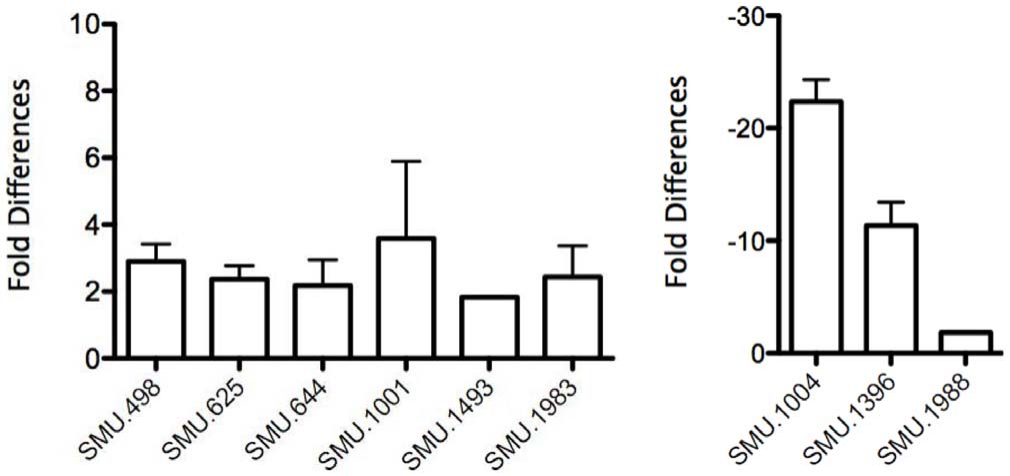
Differential regulation of gene expression measured by quantitative real-time PCR. Bars represent either repression (A) or activation (B) in strain IBS10 (*covR*) relative to the wild-type strain UA159. Values are means ± standard deviations from at least three independent experiments.

In an attempt to validate the microarray results, quantitative real-time RT-PCR analyses were carried out. Toward this end, RNA isolated from exponentially grown cultures of UA159 and IBS10 was used for RT-PCRs with primers listed in Table S1. Nine genes that were differentially regulated according to the microarray data (Table S2) were randomly selected, and their expression was analyzed. The genes chosen were SMU.498, SMU.625, SMU.644, SMU.1001 *(dprA)*, SMU.1004 *(gftB)*, SMU.1396 *(gbpC)*, SMU.1493, SMU.1983, and SMU.1988. The results are consistent with the observed microarray results (Fig. 1), and both methods strongly correlated with a coefficient of correlation value of R^2^ = 0.93 (data not shown). Thus, CovR indeed regulates the expression of these genes at the transcriptional level.

### CovR is involved in competence of *S. mutans*

**Figure 2:**
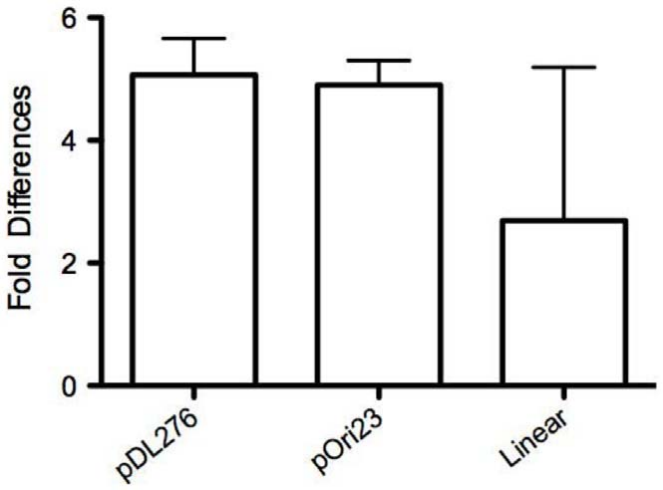
CovR is required for competence. Fold-difference in transformation efficiency of the *S. mutans* UA159 (wild-type) compared to IBS10 (Δ*covR*) with two circular plasmid DNAs (pDL276 and pOri23) and linear DNA (a PCR fragment from plasmid pIB75, see text). Mean values with standard error from two (pOri23 and pIB75) or three (pDL276) independent experiments are shown.

Our transcriptome study indicated that several competence related genes, including *coiA*, *dprA*, *comF/comFC*, *comEA/comEC*, and the genes belonging to the *comY* operon (Table S2) were down regulated in the *covR* mutant strains. The extent of down regulation varied between 1.6 to 3.6-fold. Quantitative RT-PCR confirmed that at least *dprA* was also down regulated in the *covR* mutant to a similar extent as in the transcriptome study. Thus, tit appears that a functional CovR is required for optimal competence gene expression. To determine if activation of these genes by CovR correlates with competence of *S. mutans*, both the wild-type UA159 and and Δ*covR* mutant IBS10 strains were used as recipient for the foreign DNA. Three different types of DNA molecules were used: pDL276 plasmid that replicates *via* rolling-circle mechanism; pOri23 plasmid that replicates *via* theta mechanism; and a linear DNA fragment generated by PCR that contains an erythromycin resistance marker flanked by ∼0.5-kb homology corresponding to the SMU.261 locus (a locus unlinked and unrelated to CovR). Both of the pDL276 and pOri23 plasmids were able to replicate in *S. mutans*, providing resistance to kanamycin and erythromycin, respectively, whereas the PCR product encoding erythromycin resistance (Table S1) could provide this phenotype only after integration into the chromosome. In the latter case, in addition to the defect in transformation, possible deficiencies in the recombination pathway could also be detected. As shown Fig. 2, in all cases, the transformation efficiency of the IBS10 strain was lower compared to the wild- type strain. A reduction of ∼2.7-fold was obtained with transformation of the linear DNA fragment, while a reduction of ∼5.0-fold was obtained for the replicating plasmid transformations. Thus, we conclude that CovR plays an important role in the competence of *S. mutans*.

### CovR regulates expression of the *smt* locus

**Figure 3:**
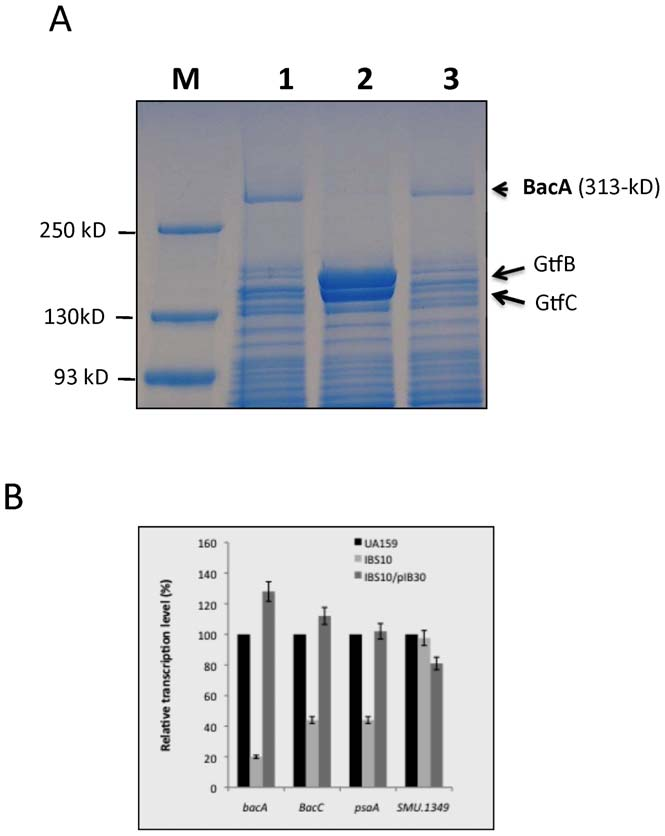
Differential expression of *smt* operon in the CovR deficient strain. (A) Protein profile of the wild-type UA159, the Δ*covR* mutant IBS10, and the complemented IBS10 strain. Whole cell lysates from cultures grown to the mid exponential phase were resolved on a 4–20% gradient gel, and stained with PageBlue (Fermentas). M: Prestained molecular weight marker; lane 1: UA159 (wild-type); lane 2: IBS10 (*covR* mutant); and lane 3: IBS10/pIB30 (complemented *covR* mutant). The 313-kDa protein band was excised from the gel and analyzed by mass spectrometry to verify its identity. (B) RT-PCR analysis of the genes in the *smt* locus. RNA was harvested from cultures at the mid-exponential phase of growth and subjected to real time RT-PCR analysis using primer pairs specific for *bacA*, *bacC*, *gyrA*, *psaA*, and SMU.1349 genes, as described in the text. Strains used include: UA159 (wild-type), IBS10 (*covR* mutant), and IBS10/pIB30 (complemented *covR* mutant). Real time RT-PCR reactions were performed in triplicate and the mean values with standard deviations are shown.

In an earlier study we also observed that a high molecular weight protein, which migrated to a point above the 175-kDa marker, was specifically expressed in the wild-type UA159, but was absent in the isogenic Δ*covR* mutant IBS10 (see Fig. 1 of reference [18]. To confirm our previous observation, protein profiles of the wild-type (UA159) and the *covR* mutant IBS10 were resolved using a 4–20% SDS-PAGE. As shown in Fig. 3A, in the crude cell lysates prepared from the exponentially growing cultures, a band above the largest marker (250-kDa) appears to be expressed in the wild-type strain, but is absent in the crude lysates of IBS10. This band was also visible in the stationary phase culture lysates from UA159, but not in the lysates of IBS10 (data not shown). To identify this band, the band was excised from the gel, and the protein was analyzed by mass-spectrometry. Twenty peptide fragments were identified by mass-spectrometry analysis, and a BLAST search revealed that the peptides corresponded to BacA (SMU.1342), which is encoded by *smt* locus within the TnSmu2 GI. To verify that the expression of the BacA protein was indeed up-regulated by CovR, total cellular protein was also extracted from IBS10 carrying pIB30, a plasmid containing full-length wild-type *covR* [18], and its protein profile was compared with the wild-type UA159 and Δ*covR* mutant strain IBS10 (Fig. 3A). The profile of the complemented *covR* mutant strain displayed the same band as the wild-type strain, suggesting that the loss of the BacA protein in crude cell extract of IBS10 was due to the inactivation of *covR*.

The transcriptome study suggested that CovR positively regulates all ten genes belonging to the *smt* locus. The absence of BacA protein in the Δ*covR* mutant strain is consistent with the microarray result. To confirm the effect of CovR on the transcription of *smt* locus, we performed real time RT-PCR analyses using RNA isolated at mid-exponential growth phase from strains UA159, IBS10, and IBS10/pIB30. In order to measure the level of the transcripts produced from each strain, RT-PCR was performed using specific primers corresponding to the following three genes, *psaA* (SMU.1348, the first gene in the *smt* operon), *bacA* (SMU.1342), and *bacC* (SMU.1339, the last gene in the *smt* operon). The level of the *gyrA* transcript was also measured to ensure that equal amounts of RNA were being used for each RT-PCR reaction. The *bacA* transcript produced from IBS10 grown to mid-exponential phase was one-fifth compared to the wild-type UA159 strain (Fig. 3B). The complemented Δ*covR* mutant strain, IBS10/pIB30, produced about 1.2-fold more *bacA* transcript compared to the wild-type UA159. Similarly, both the *psaA* and *bacC* transcripts were also reduced in the *ΔcovR* mutant strain, where the transcript levels were about 44% compared to those of the wild-type strain (Fig. 3B). As expected, the expression of SMU.1349, which lies just upstream of the *smt* operon, was not significantly altered (Fig. 3B). We also measured the relative level of the *bacA* transcript from cultures grown to stationary phase and found that it followed the same pattern as the level in the mid-exponential phase (data not shown). Taken together, our results suggest that when CovR is present, expression of the entire *smt* locus, including the *bacA* gene, increases significantly.

### CovR binds to promoter regions of both activated and repressed genes

**Figure 4:**
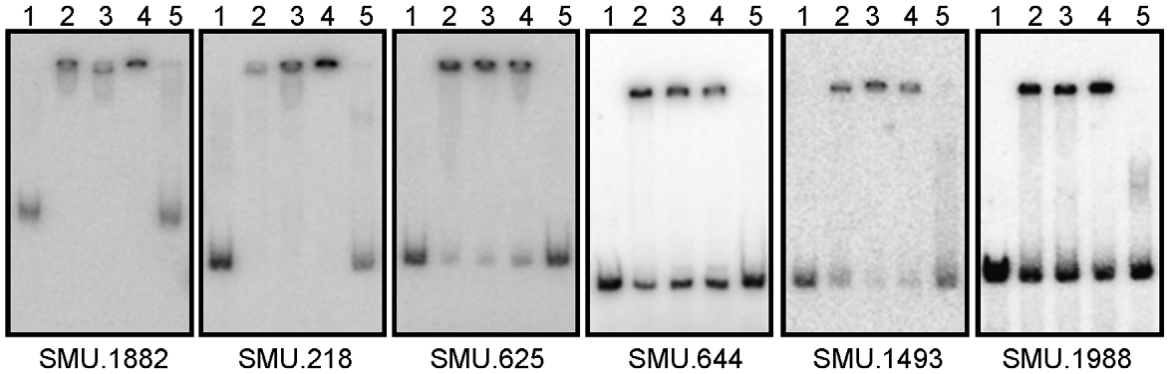
Binding of CovR to the putative promoter regions of the genes indicated below the respective panels. Increasing concentrations of CovR was added to 0.1 pmole of the putative promoters as follows, lane 1, 0 µM; lane 2, 0.5 µM; lane 3, 1.25 µM; lane 4, 2.5 µM. Lane 5 contains 1.25 µM CovR with 10 pmole of non-labelled specific DNA.

Our earlier studies demonstrated direct binding of CovR to promoters of four repressed genes and one activated gene. To further investigate the interaction of CovR with the regulated promoters, a total of 15 promoters were subjected to electrophoretic mobility shift assays (EMSA) with purified His-CovR protein. Among the promoters, CovR repressed four of them while the rest were activated by CovR (promoters used for EMSA are indicated in Table S2). For all 15 promoters except one, shifts in the mobility were observed when CovR was added, indicating direct binding of CovR to the DNA sequences upstream of both CovR-activated and -repressed genes (Fig. 4, data not shown). We did not observe any shift after incubation of CovR to the SMU.136 promoter, suggesting that regulation of this gene by CovR is indirect. We also did not observe any shift when CovR was added to a DNA fragment corresponding to the *nlmAB* operon, a result consistent with the absence of CovR regulation of this locus and allowing for this DNA fragment to be used as a negative control for the specificity of CovR binding to regulated promoters. Specificity of the binding interaction for each of the regulated promoters was also supported by competition with excess unlabeled probe (Fig. 4, lanes 5). Therefore, CovR specifically binds to the target promoters to activate or repress transcription of these genes, and supports the results of our microarray analysis.

### Inactivation of *covR* perturbs protein expression

**Figure 5:**
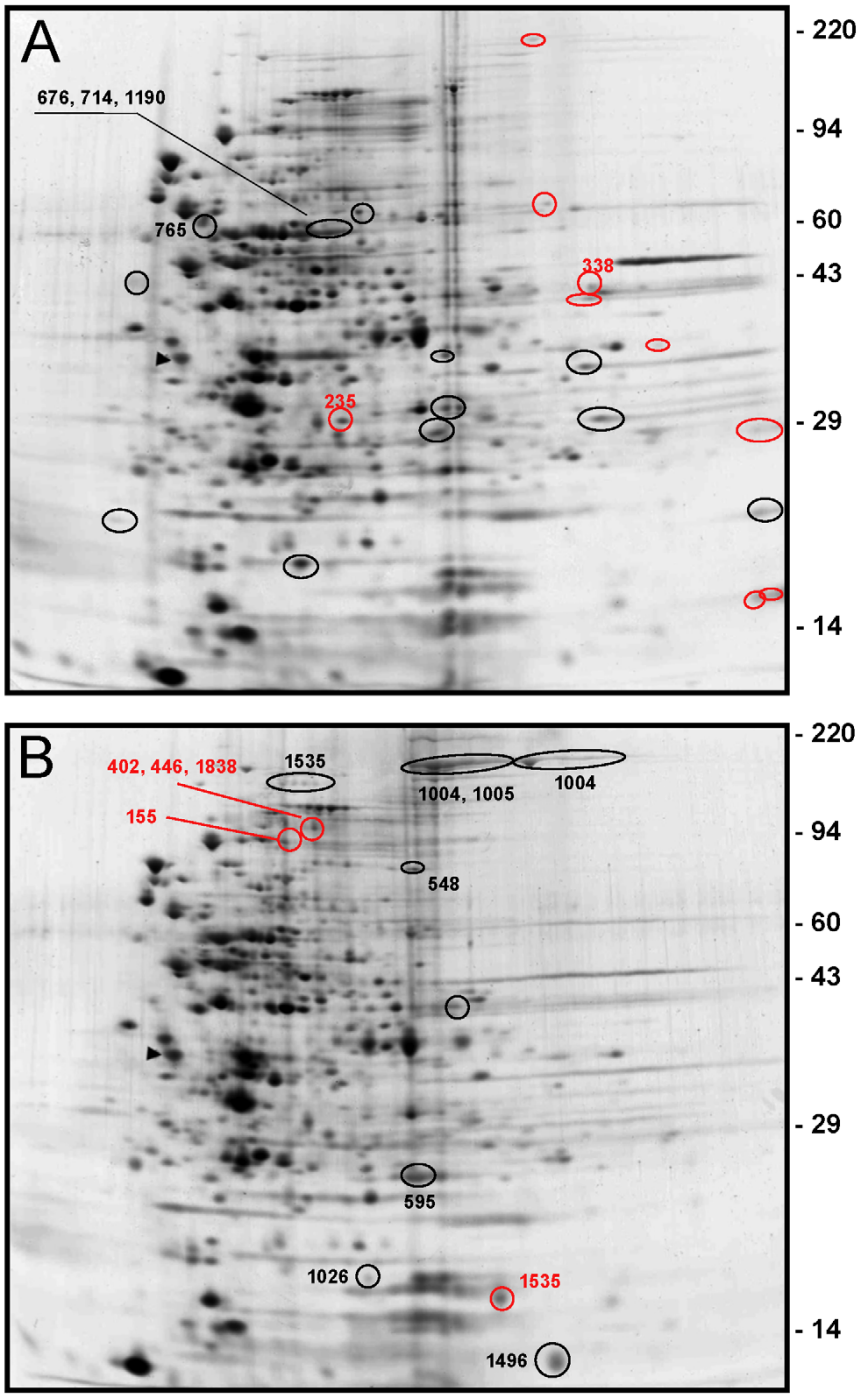
Two-dimensional gel electrophoresis analysis of *S. mutans*. Whole cell lysates of the wild-type UA159 (A) and Δ*covR* mutant IBS10 (B) are electrophoresed in 10% SDS-PAGE. Red and black circles in Fig. 2A indicate protein spots exclusively present in wild-type strain, or at least 3-fold abundant in the wild-type strain compared to the *covR* mutant, respectively. Red and black circles in Fig. 2B indicate protein spots exclusively present in the *covR* mutant strain, or at least 3-fold abundant in *covR* mutant strain compared to wild-type strain, respectively. Numbers correspond to the orf according to NCBI designation (SMU#). Molecular weight standards (kD) are shown.

Our one-dimensional SDS-PAGE analysis showed that at least three differentially expressed proteins (GtfB, GtfC, and BacA) could be easily detected, distinguishing the wild-type UA159 from the *covR* mutant IBS10 (Fig. 3A, [18]). To evaluate a possible relationship between transcriptional and translational regulations that is modulated by CovR, a two-dimensional gel electrophoresis approach was used to identify the protein spots that are differentially expressed in UA159 and IBS10. Crude cellular lysates from UA159 and IBS10 were prepared from mid-exponential-phase and subjected to proteomic analysis. A total of approximately 480 polypeptide spots could be detected in the pI range of 5.2 to 8.2 by silver staining (Fig. 5, data not shown). Three-fold difference in spot densities was used as cut-off to identify differentially expressed proteins/protein isoforms in the UA159 and IBS10 strains. Comparison of the proteomes from UA159 and IBS10 revealed that at least 41 protein spots had levels of expression altered by a change of three-fold or greater, with a P- value of ≤0.05. Among the differentially expressed proteins, 26 were down regulated while 15 spots were up regulated in IBS10 strain relative to those in the wild-type strain grown under the same condition. Fourteen spots of interest were excised from the gels, and the polypeptides were identified by mass-spectrometry. Corresponding protein identities are indicated in the Fig. 5 by their SMU# according to the GenBank *S. mutans* genome annotation [15]. For example, the spot identified as SMU.235 (hypothetical protein) was exclusively present in the UA159 strain, while the spot identified as SMU.155 (polynucleotide phosphorylase) was exclusively present in the IBS10 strain. On the other hand, the spot identified as SMU.1496 (galactose-6-phosphate isomerase) was present in both the strains, but was 3-fold more abundant in the Δ*covR* mutant. In total, 18 peptides present in the 14 spots were unambiguously identified by mass-spectrometry. Overall, apart from GtfB and GtfC, protein expression pattern did not correlate with the microarray results.

### CovR modulates FTF expression

**Figure 6:**
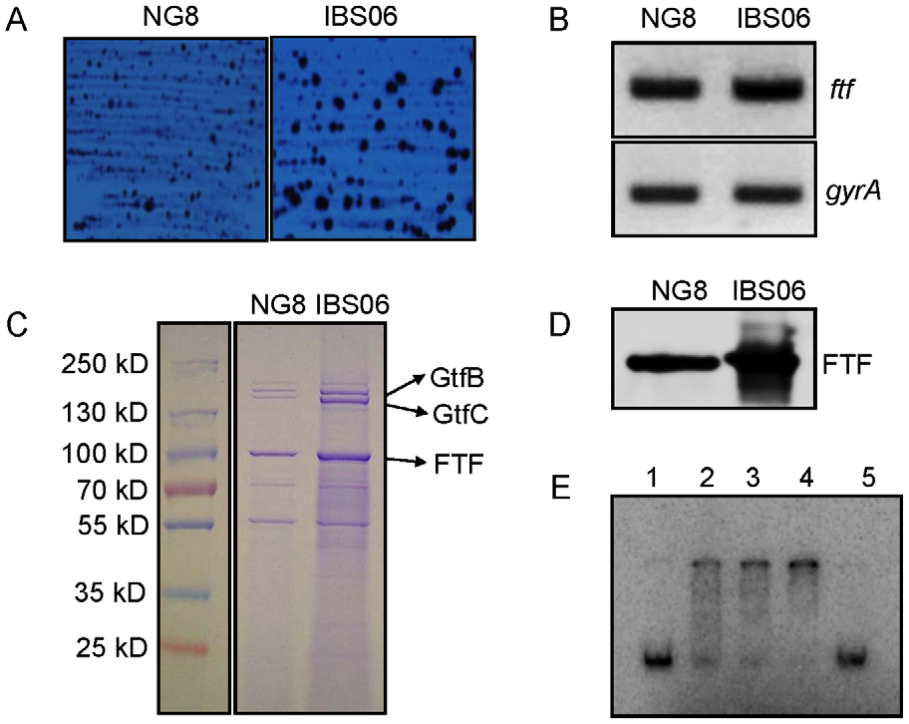
Differential expression of fructosyltransferase in wild-type and Δ*covR* strains of NG-8. (A). Colony morphology of IBS06 (*covR* mutant) and NG-8 (isogenic wild-type parent) on mitis-salivarius agar medium. Plates were incubated at 37°C under microaerophilic conditions for 48 hrs. (B) Semi-quantitative RT-PCR analysis of *ftf* and *gyrA* for the strains NG-8 and Δ*covR* (IBS06). The *gyrA* gene was included as an internal control to ensure that equal amounts of RNA were used for each RT-PCR reaction. Experiments were repeated at least twice with two independent RNA isolations. (C) Analysis of extracellular proteins from the wild-type and the Δ*covR* strains. Supernatant proteins from overnight cultures were precipitated by 20% TCA, washed with acetone, and resuspended in PBS. Equal amounts of protein were loaded in each lane, and samples were run on SDS-PAGE (4–20%) gels and stained with Coomassie blue. Bands marked with arrowheads were excised from the stained gel, and identified by mass spectrometry. Lanes: M, Fermentas prestained marker; 1, NG-8; 2, IBS06. Proteins identified by mass spectrometry are indicated at the right. (D) Western blot analysis of FTF (fructosyltransferase) expression. NG-8 (wild-type, lane 1) and IBS06 (*covR*, lane 2) were grown overnight in THY broth and whole-cell extracts were prepared. Equal amounts of cell extracts were separated on 4–20% SDS-PAGE gels and reacted with anti-FTF antibody (E). *In vitro* binding of CovR to the promoter of *ftf* (P*_ftf_*). EMSA was performed with His-tagged CovR as described in the text. An increasing concentration of CovR was added to 0.1 pmole of the putative promoters as follows: lane 1, 0 µM; lane 2, 0.5 µM; lane 3, 1.25 µM; lane 4, 2.5 µM. Lane 5 contains 1.25 µM CovR with 10 pmole of non-labelled P*_ftf_* DNA.

Our transcriptome data suggest that one of the virulence-related genes, *ftf* (SMU.2028), was moderately up-regulated in the *covR* mutant strain. FTF, which synthesizes fructan polymers from sucrose, can be found in either cell-associated or extracellular form. Expression of FTF varies greatly among various *S. mutans* isolates. Although FTF can be identified in UA159, it appears that FTF is one of the most abundant proteins in the culture supernatant of NG-8 [48]. Therefore, we chose to investigate the role of CovR in the production of FTF in this strain, and constructed a *covR* mutant strain, IBS06. IBS06 appeared to produce heavily mucoid colonies compared to NG-8 when streaked on mitis-salivarious agar plates (Fig. 6A). The extracellular protein profiles of the wild-type (NG-8) and the Δ*covR* mutant (IBS06) were also determined by resolution *via* 4–20% gradient SDS-PAGE. Two distinct bands of approximately 170-kDa were elevated in the Δ*covR* mutant IBS06 relative to the wild-type NG-8 strain. Mass-spectrometry analysis confirmed that the larger band corresponds to GtfB, while the smaller band corresponds to GtfC. As previously shown, these two bands were also up regulated in IBS10 supernatant fractions (Fig. 3A). In contrast, the supernatant fraction of NG-8 and its isogenic *covR* mutant strain contain a band that was absent in UA159 and its derivatives. This band, which is ∼87.0 kDa in size, was more abundant (∼3-fold) in the mutant strain (IBS06) compared to the wild-type strain (NG-8). Mass-spectrometry analysis confirmed the identity of the band as FTF protein. To verify our mass-spectrometry result, we employed western blot analysis (Fig. 6D). Mid-exponential phase culture supernatants from the wild-type (NG-8) and the *covR* mutant (IBS06) strains were separated by SDS-PAGE, and probed with anti-FTF antibody. As expected, IBS06 showed increased presence of FTF in the supernatant compared to NG-8. Furthermore, purified CovR protein was able to bind to the promoter region of the *ftf* gene during EMSA analysis (Fig. 6E). This binding was specific, since excess unlabelled competitor DNA was able to disrupt the complex formation. Thus, taken together our results strongly suggest that in addition to glucosyltransferases (GtfB/C), CovR also regulates fructosyltransferase production in *S. mutans* strain NG-8.

### CovR is involved in regulation of multiple genes in NG-8

Since our SDS-PAGE analysis of the supernatant proteins identified at least three genes that were up-regulated in the *covR* mutant IBS06, we investigated the CovR regulon in NG-8, by measuring the expression of some of the selected genes that were regulated by CovR in its sister strain UA159. We found that *gtfB* (SMU.1004), *gtfC* (SMU.1005), and *gbpC* (SMU.1396) were all up regulated 3-fold or higher in strain IBS06 as compared to the parental strain NG-8. We also found that the putative galactose metabolism operon (SMU.1431–SMU.1437) and a hypothetical protein (SMU.2147) were also up regulated in the *covR* mutant. Surprisingly, we found that the operon encoding genes SMU.1067 to SMU.1070 (putative ABC transporter genes) was also up-regulated in the IBS06 strain. This operon was not differentially regulated in UA159 or its isogenic *covR* derivative IBS10. Among the CovR-activated genes, we measured the expression of SMU.1882 gene, and found that this gene was down regulated ∼3.0-fold in the *covR* mutant strain IBS06 as compared to the wild- type parent (NG-8). Unexpectedly, we found that the operon encoding citrate utilization genes, which also includes a transporter gene (SMU.1010–SMU.1013), were also down- regulated (∼2.0-fold) in IBS06. Since both TnSmu1 and TnSmu2 are absent in NG-8 (Biswas, unpublished), we were unable to test the expression of the genes associated with these operons.

## Discussion

CovR/S is an important two-component signal transduction system that seems to regulate a wide range of genes in various Streptococcal *spp*. Although it has been known for pathogens such as GAS and GBS that CovR/S plays a fundamental role in virulence and stress response, the global role of this system in *S. mutans* biology and/or pathogenesis has not been elucidated in detail. In contrast to other streptococci in which this TCS system is present, CovR appears to be an orphan response regulator in *S. mutans* [20], [21], [24], [27], [29], [35], [49]. To understand the importance of this response regulator, we carried out detailed genomic and proteomic studies. We report here that under planktonic growth conditions, CovR plays pleiotropic and complex roles in *S. mutans* global gene regulation, including competence and expression of important virulence traits. Our analyses have revealed several new findings that are discussed below.

Previous studies from our laboratory indicated that CovR predominantly functions as a transcriptional repressor. We characterized four such genes: *gtfB*, *gtfC*, *gbpC*, and *covR*; expression from each of these promoters is repressed by CovR [17], [18], [33]. We also demonstrated that CovR could activate expression of SMU.1882, a small putative bacteriocin-encoding gene. However, in this present study, our transcriptome data suggested that among the 128 differentially regulated genes (about 6.5%), 69 genes were activated, whereas 59 genes were repressed by CovR. Although the number of genes regulated by CovR in *S. mutans* is smaller compared to GAS (6.5% vs 15%), it is comparable to the number of differentially regulated genes in GBS (∼7%). Our results are also consistent with the GBS data that show the number of CovR activated and repressed genes are roughly equal. In contrast, CovR activates very few genes in GAS [21].

Among the 41 protein spots that were differentially expressed in our proteomic study, the identity of only 14 protein spots were unambiguously determined (Fig. 5). The expression of GftB (SMU.1004, glucosyltransferase-I) and GtfC (SMU.1005, glucosyltransferase-SI) were more than 3-fold higher in the *covR* deficient strain IBS10, and correlated with the microarray analysis. However, expression of other proteins (or protein isoforms) identified during the proteomics analysis did not correlate with the microarray results. There are several possibilities that can explain the lack of correlation between the transcriptomics and proteomics data. First, the results of microarray analysis are more comprehensive compared to those obtained in the proteomics study. Furthermore, due to the relatively low sensitivity of protein detection, the total number of unique proteins identified with 2-DE followed by mass-spectrometry usually corresponds to about 10% of the predicted proteins within the range of pH gradient [50], [51], [52], [53]. The low abundance proteins, such as transcriptional regulators, are difficult to detect in the proteome, although transcriptional levels of the corresponding genes can be easily measured by microarrays. Furthermore, many streptococcal proteins are known to be expressed as different isoforms; the data presented here also confirm this. For example, among the 14 spots of interest, two spots identified in the *covR* mutant contained SMU.1535 (glycogen phosphorylase). One spot was exclusively found in the *covR* mutant, while the other spot was more abundant in the mutant compared to the wild-type strain (Fig. 5B). We cannot rule out the possibility that SMU.1535 is present in the wild-type proteome, and based on our limited analysis of just a few protein spots it is difficult to conclude that CovR regulates expression of all the identified protein spots (Fig. 5). Nevertheless, given that the proteome maps were well reproducible, and a total of 41 protein spots were differentially expressed, it is obvious that inactivation of *covR* resulted in some perturbations at the translational and/or post-translational level.

Among the new findings that emerged from this study is the involvement of CovR in the regulation of competence-related genes, where CovR appeared to activate at least 16 competent related genes. Among these, four genes encode late competentence proteins (ComEA, ComEC, ComFA, and ComFC) that are involved in DNA uptake. It was found that CovR also activated genes belonging to the ComY operon as well as a gene encoding DprR protein. This protein, which colocalizes with the DNA uptake machinery [54] in streptococci, has two functions. The first function is single-strand DNA binding activity that is absolutely required during DNA uptake, and the second activity is to sequester intracellular iron to prevent H_2_O_2_ toxicity. Consistent with the gene expression studies, we found that *covR* mutant strains are more sensitive to H_2_O_2_ exposure in a disk diffusion assay (data not shown). Our results are also consistent with the results in *S. pneumoniae*, where *dprA* is also activated by RitR, an orphan response regulator that shares a high degree of sequence similarity to CovR [55]. However, in this pathogen, RitR does not activate other competence-related genes. Our microarray results were also supported by the transformation studies, which found three- to five-fold reduction of transformation efficiency in the *covR* mutant.

The CovR/S system also actively participates in general stress responses in both GAS and GBS. In the case of GAS, a functional system is necessary to withstand acid, osmotic, and thermal stresses. CovR/S system is involved in acid stress response in GBS. More than 90% of the pH-activated genes are also regulated by CovR/S [56]. Surprisingly, we did not find any correlation between acid-modulated and CovR-regulated genes. However, we found several oxidative-stress related genes that appeared to be activated by CovR, although the observed activation is low to moderate (1.5- to 2.0-fold). Further experimental verification is required to confirm the role of CovR in the oxidative stress response in *S. mutans*.

Animal studies have indicated that both GAS and GBS CovR plays an important role in pathogenesis. However, the effect of CovR on relative virulence depends on the experimental animal models and strain types. For example, CovR deficient strains of GAS are shown to be hypervirulent in a murine skin infection model [57]. However, a naturally occurring *covR* mutant strain of GAS has also been shown to be hypervirulent in a murine model of infection [58]. The role of CovR in GBS pathogenesis is much more complex, and highly strain dependent [27]. Streptococcal pathogenesis has also been studied using zebrafish as an experimental animal model system [59]. Our preliminary studies also indicate that IBS10, the isogenic *covR* mutant derivative of UA159, is also hypervirulent in a zebrafish infection model as compared to UA159 (Neely and Biswas, unpublished).

Another important finding is the involvement of CovR in the regulation of genes that are encoded within the genomic islands (GIs). Out of 11 GIs that are encoded by the UA159 genome [15], we found that genes encoded by two GIs were differentially expressed in the Δ*covR* strain (Table S2). GI-6, which is also known as TnSmu1 [46], encodes about 34 genes, and we found that 13 of these genes were up-regulated in the Δ*covR* strain. Only one gene, SMU.223, which is also encoded by TnSmu1, was down-regulated in the mutant. On the other hand, at least 12 genes encoded by GI-12, also known as TnSmu2 [45], were down-regulated in the Δ*covR* strain. Interestingly, the difference in the expression of these genes between the wild-type and the mutant was among the highest. TnSmu2 encodes an operon, *smt*, which is thought to be involved in the biosynthesis of secondary metabolites. There are ten genes (SMU.1339–SMU.1348) encoded within the *smt* operon, and all of the genes were down-regulated in the *covR* deletion strain. Furthermore, one gene product of the *smt* operon, BacA, was also identified by the proteomics analysis. However, the expression of SMU.1349, a TetR/AcrR family of transcription factors, which lies just upstream of the *smt* operon, was unaffected in the *covR* deletion strain. At present, we do not know the exact mechanisms by which CovR modulates the expression of these GI encoded genes. It is possible that CovR may activate the *smt* operon by functioning as an anti-silencer.

By using DNase I protection assays, previously we have characterized the binding of CovR to promoters of five genes: *gtfB*, *gtfC*, *gbpC*, *covR*, and SMU.1882 [17], [18], [33]. These five promoter sequences have been analyzed with the GLAM2 program to develop an optimized position weight matrix for consensus binding sequence (CBS) for CovR [17], [18], [33]. Surprisingly, four different CBSs have been obtained with overall weight score varied from 78.4 to 67.8. The CBS with the highest score (78.4) is a 36-bp long AT-rich sequence that is present in all the five promoters; in one promoter (*gtfC*), the sequence is present twice. When we analyzed the presence of this putative CBS in the *S. mutans* genome, we found the sequence was present about 25 times, many of which map to the intergenic regions (IGR); however the majority of the IGR were not associated with the CovR regulon. In this study, we extended the binding study to 15 additional promoters. With one exception, CovR was able to bind to all of the other tested promoters. To develop a revised CBS, we analyzed by GLAM2 all 19 promoters to which CovR was shown to bind directly. Apart from the presence of a few AT-rich motifs at the binding sites of these promoters, we were unable to derive a consensus binding sequence specific for CovR. Furthermore, as mentioned before, the GAS CovR consensus binding sequence, ATTARA [29], [30], was not found in all of the promoter sequences regulated by *S. mutans* CovR. Since no specific binding sequence is apparent for *S. mutans* CovR, it is possible that CovR may have low sequence specificity for DNA binding. Alternatively, CovR of *S. mutans* may recognize and bind to multiple sequences that cannot be deduced by a simple sequence comparison. It is also possible that CovR recognizes some unique structural features in the target DNA, such as bending or looping. Further *in vitro* experiments are required to elucidate the mechanism by which CovR recognizes its target promoters.

Homologs of the CovR/S system are widely present in many streptococci. However, in most cases this is an archetypal system that includes both the response regulator (CovR) and the histidine kinase (CovS). In the case of *S. mutans*, CovR appears to be an orphan response regulator [17], as no CovS homolog can be identified in this pathogen, although the locus is highly conserved in both GAS and GBS, as well as *S. thermophilus*. The exact environmental cues that stimulate this system are also unknown, although Fe^+^, Mg^+2^, cationic peptides, and environmental stresses have been shown to activate this TCS [36], [60], [61]. We have shown that *covR* expression in *S. mutans* is dependent on temperature, pH, as well as Mg^+2^; however whether these signals truly activate CovR *in vivo* remains to be evaluated.

The precise mechanism in the activation of CovR/S system is not fully understood. In the case of GAS, it has been shown that CovS acts both as a phosphatase and as a kinase. While phosphorylation of CovR causes dimerizarion, and possibly recruitment to the target promoters, dephosphorylation by CovS leads to inactivation of CovR. Depending on the specific environmental signal, the activity of CovS is switched between phospahatase and kinase. On the other hand, in GBS, eukaryotic-like Ser-Thr protein kinase-phosphatase (STK-STP) pairs have been implicated in the regulation of CovR activity. In this case, CovR is proposed to be phophorylated at two residues: an aspartate at position 53 (D53), and a threonine at position 65 (T65). While phosphorylation of CovR at D53 by CovS leads to activation of the system, phosphorylation of CovR at T65 by STK leads to inhibition [62]. Thus, two different kinases modulate the activity of CovR in GBS. The involvement of two kinases seems to be specific to GBS, since CovR is not modulated by STK in GAS. For three reasons, we believe that CovR activity is also not modulated by STK in *S. mutans*. First, unlike the GBS scenario, there is very little overlap between the STK and the CovR-regulated genes. Second, the conserved T65 residue is absent in the *S. mutans* CovR protein. Finally, an *in vitro* phosphotransfer reaction failed to transfer the phosphate group from STK to CovR (data not shown). Since CovS is absent in *S. mutans*, we speculate that CovR is engaged in cross talk with other TCSs. Furthermore, small molecule phosphodonors, such as acetyl phosphate, may play an important role in the modulation of CovR activity in *S. mutans*. Further biochemical studies are required to unravel the mechanism of CovR activation in *S. mutans*.

## Supporting Information

Table S1 List of oligonucleotides used in this study.
(DOC)

Table S2 Transcriptome changes associated with *covR* inactivation in *S. mutans* UA159 (1.5-fold or greater with P<0.05).
(DOC)
