# Supplementary material for: CovR-Controlled Global Regulation of Gene Expression in Streptococcus mutans
Source: PLoS One. 2011 May 31;6(5):e20127. doi: 10.1371/journal.pone.0020127 (PMC3105014; doi:10.1371/journal.pone.0020127)
Supplement: Table S1 — List of oligonucleotides used in this study. (DOC) [file pone.0020127.s001.doc]

**Table S1: List of oligonucleotides used in this study**

**-----------------------------------------------------------------------------------------------------------------------------------------**

**Primer Sequence (5'-3') Purpose**

**-----------------------------------------------------------------------------------------------------------------------------------------**

Smu. 498F TATTATGGCCGCCTCTTGACCG Real Time PCR

Smu. 498R GCCGCAACGATTGCAGATAAAG Real Time PCR

Smu. 625F TGGCTCGTTTAAAGGAACAATCCC Real Time PCR

Smu. 625R GCAGTCGTCTGCGTCTTCTTCTGA Real Time PCR

Smu. 644F AGTGATTTTTATTGTCCTGCCTGCC Real Time PCR

Smu. 644R CAAAATGCGGACGCATAACCCT Real Time PCR

Smu. 1001F GCAACGCCAGTCAAATGGGAGT Real Time PCR

Smu. 1001R GTATCAATACCACGCGCTAAACCG Real Time PCR

Smu. 1004F CTGTTGGGGAAACAGCTTCTAATGG Real Time PCR

Smu. 1004R GCAGCAGCTTCAGAAGTTTTGTCAA Real Time PCR

Smu. 1005F TAAAAGCAGATTCAACTGACGACCG Real Time PCR

Smu. 1005R GTTTCTTTTGCTGCTTCACTTGTCG Real Time PCR

Smu. 1339F AACCTCAAAATAAAATCGAAGAAACGC Real Time PCR

Smu. 1339R GCTGTGGAAGAAGGATTAAATAGCTGA Real Time PCR

Smu. 1342F GGGGCAAAGAATTATCAATTGAAGCTCCAACC Real Time PCR

Smu. 1342R GCATTTGAAAATTGATCTACTTCAACTGGAGC Real Time PCR

Smu. 1348F CAGGAAATTGAAGCAGTAAAGCATG Real Time PCR

Smu. 1348R CCCTTTGTTGTTCACCTCCTGA Real Time PCR

Smu. 1396F CCTTGCTTTGGCGGCTTTTG Real Time PCR

Smu. 1396R GCCGGTTCTGATGCTTGTGTATCA Real Time PCR

Smu. 1398F GGCTATTTCTGCCAATCTTCAAA Real Time PCR

Smu. 1398R ATCCTCAGTCGTTTCATCACTG Real Time PCR

Smu. 1983F CATGTCGCGAATTAAAGCTTTTACG Real Time PCR

Smu. 1983R CGCTCTTCTACTTTGGCAAACGTTT Real Time PCR

Smu. 1988F ACCGTGGTGGTTTTTAGATGATTGG Real Time PCR

Smu. 1988R GCCCACTCTTTTTGATAACACTGCA Real Time PCR

Smu gyrA F CTATGAGTGTTATTGTTGCTCGGGCT Real Time PCR

Smu gyrA R CCCCCAATTCATTCATGCCATATA Real Time PCR

Smu.2028 RT F GAGAATGGAGATCGCTATCTTGTCTTTGA Semi-quantitative RT PCR

Smu.2028 RT R CCAAGCATCGTTGTTACTTCCGTGA Semi-quantitative RT PCR

GbpC-F4 GAGAAAGCACTTTTGGTTTCAATTGGAAC Gel Shift Assay

GbpC-Fout2 GGCTGCTTAGCAAAGTAATTTTAGCAGTTTTCG Gel Shift Assay

Smu. 136 Pr F GTGAGGGGGCAAAAGACCAATAAGTC Gel Shift Assay

Smu. 136 Pr R AATTAAAGAATACAAGTTTAAA Gel Shift Assay

Smu. 217 Pr F CCCTCTATCATATTTTTTCCCCTATTCT Gel Shift Assay

Smu. 217 Pr R AGGCGGTAAGCTAGTCTATAACGAGTGA Gel Shift Assay

Smu. 218 Pr F ACGCACTTTTTTATTCAATAAT Gel Shift Assay

Smu. 218 Pr R CTGGGAACATATTTGATCCTT Gel Shift Assay

Smu. 498 Pr F GCTGAGCATGAATGTCGTGCCTT Gel Shift Assay

Smu. 498 Pr R CTCCATCACCTTTCTTATTCGTAAACA Gel Shift Assay

Smu. 609 Pr F GGAAGAGAAGATGTAACAATGA Gel Shift Assay

Smu. 609 Pr R GCTTGATGATTAATATCAATTC Gel Shift Assay

Smu. 625 Pr F TGGCTATTGTTCTTGCTTTC Gel Shift Assay

Smu. 625 Pr F CGTCAACCATTTTAACCTCCTT Gel Shift Assay

Smu. 644 Pr F CCGCTGTTTAAAGTGCTGACTA Gel Shift Assay

Smu. 644 Pr R CCCTTGCAACTAACATCAAA Gel Shift Assay

Smu. 670 Pr F GCCATTTTTTCCTCCTAAATTT Gel Shift Assay

Smu. 670 Pr R GCCGATTCAACGTAAAAGTTGA Gel Shift Assay

Smu. 941 Pr F GCAGAGATGGTATTTTTTCTATTCCTCA Gel Shift Assay

Smu. 941 Pr R CTTGATTGGCTTTTCAATATGACCATGT Gel Shift Assay

Smu. 1001 Pr F GGGATATTGTTTTTATTTTTGGGAGTCGCT Gel Shift Assay

Smu. 1001 Pr R GTTGAAAATTATCCATTTTTATACCTCACT Gel Shift Assay

Smu. 1398 Pr F AGTCGGATCCTACCTAAAAATAGCAGTATT Gel Shift Assay

Smu. 1398 Pr R CTCTCTCGAGAGAAATAGCCTTCATTGCTTC Gel Shift Assay

Smu. 1496 Pr F GAGCGAGAGAAGACCTTCAAGATTGTTTAG Gel Shift Assay

Smu. 1496 Pr R CCAGCTGCATCTGAACCAATAATAATTG Gel Shift Assay

Smu. 1882 F2 CAGCTATACTGCTGAGGCTCCCTAGATC Gel Shift Assay

Smu. 1882 R2 GTAGCTTTAAAATCTTCAAACGCTTGAC Gel Shift Assay

Smu. 1985 Pr F GGGAGGAGGGATTATTGACTTTGCC Gel Shift Assay

Smu. 1985 Pr R TGCAGACCATTCAACAACCCTTGTC Gel Shift Assay

Smu. 1987 Pr F GTGGTGACTATGTACAGCGGTATCATTCTC Gel Shift Assay

Smu. 1987 Pr R CAGCTTCGGCAATGATTTTTCTACCTAATT Gel Shift Assay

Smu. 1988 Pr F CCGGCTGGTACTGGTATGGCGC Gel Shift Assay

Smu. 1988 Pr R CTTGATACATAGTTTTATTTCC Gel Shift Assay

IGR 1079 F ACAGCACCGCTAGGTTTTTTAGCTTGCTTA Gel Shift Assay

IGR 1079 R TTCTTACGATCATCATGTACCGCAATCA Gel Shift Assay

IGR 1588 F CGTCCTTTGCGTCGGACACTTC Gel Shift Assay

IGR 1588 R CTTGAACCTGACTTCTTTCAGCC Gel Shift Assay

---------------------------------------------------------------------------------------------------------------------------------------------------------------------------------------------
