# Supplementary material for: CovR-Controlled Global Regulation of Gene Expression in Streptococcus mutans
Source: PLoS One. 2011 May 31;6(5):e20127. doi: 10.1371/journal.pone.0020127 (PMC3105014; doi:10.1371/journal.pone.0020127)
Supplement: Table S2 — Transcriptome changes associated with covR inactivation in S. mutans UA159 (1.5-fold or greater with P<0.05). (DOC) [file pone.0020127.s002.doc]

**Table S2. Transcriptome changes associated with *covR* inactivation in *S. mutans* UA159**

**(1.5-fold or greater with P<0.05)**

**Category and SMU#^a,b^ Gene(s)^c^ Description^d^ Fold–change^e^**

____________________________________________________________________________________

**Competence**

498-499 *comFA*-*comFC* late competence proteins -(3.0, 3.2)

625*-626 *comEA*-*comEC* competence proteins -(2.8, 3.2)

644* *coiA* competence protein CoiA -1.6

836 GBS Bsp-like repeat -1.5

1001* *dprA* putative DNA-processing protein (Smf family) -3.6

1967 *ssbA* single-stranded DNA-binding protein -1.6

1979-1980-1981-1982- *ytxK*/-/*comGF*/-/ *comY* operon, natural competence -(1.6, 2.7, 2.8, 2.5,

1983-1984-1985*-1987* *comYD*/*comYC*/*comYB*/*comYA* 2.8, 2.2, 2.3, 2.3)

**Virulence**

1004 *gtfB* Glucosyltransferase I 20.3

1005 *gtfC* Glucosyltransferase SI 1.6

1091 *wapE* cell wall protein WapE 2.0

1396* *gbpC* glucan-binding protein C, GbpC 8.6

2028 *ftf* levansucrase precursor; β-D-fructosyltransferase 1.6

610 *spaP* cell surface antigen SpaP -1.5

940 *patB* hemolysin III -1.8

**Biosynthesis/metabolism**

438 putative (R)-2-hydroxyglutaryl-CoA dehydratase 2.2

activator-related protein

531 *pheA* chorismate mutase type II 1.7

858 *pyrB* aspartate carbamoyltranferase 1.7

859 *pyrA* carbamoyl-phosphate synthase small subunit 1.6

1222-1223-1224 *pyrF*/*pyrDB*/*pyrK* pyrimidine biosynthesis operon 1.5, 2.0, 2.2

1432 *bgc* putative endoglucanase precursor 2.8

1434 *icaA* putative glycosyltransferase 2.4

1437 *yvyH* putative UDP-N-acetylglucosamine 2-epimerase 2.7

670*-671-672 *citB*/*citZ*/*idh* glutamate biosynthesis operon -(2.0, 2.0, 2.0)

886 *galK* galactokinase -1.9

1493-1494-1495-1496* *lacD*/*lacC*/*lacB*/*lacA* galactose metabolism operon -(1.5, 1.7, 1.7, 1.9)

2127 *gabD* succinate semialdehyde dehydrogenase -1.6

**GI Associated**

199-200-202-204-205-206 hypothetical protein 1.7, 1.5, 1.7, 1.7,

1.8, 1.7

208 transposon protein, DNA segregation ATPase 1.6

209-210-211-212-213-215 hypothetical protein 1.7, 1.7, 1.7, 1.7,

1.6, 1.5

217* conserved hypothetical protein 1.8

218* transcriptional regulator, HTH-XRE family

like proteins 1.8

1352-1353 putative transposase 1.8, 1.7

100 *sorD* sorbose PTS system, IIB component -2.1

223 hypothetical protein -1.7

1339-1340-1341-1342- *bacD*/*bacC2*/*grs*/*bacA1*/ antibiotic biosynthesis operon -(8.4, 9.8, 10.3, 10.1,

1343-1344-1345-1346- *pksC*/*fabD*/*ituA*/*bacT*/ 12.8,13.7,17.1,13.1)

1347-1348 *ylbB*/*psaA* -(8.1, 8.0)

1365-1366* *ylB*/- putative ABC transporter, permease protein, -(7.6, 8.0)

ABC transporter; ATP-binding protein

**Stress Response**

758 NADH dehydrogenase (ubiquinone) 2.4

1322 *budC* acetoin reductase 1.6

1406 putative oxidoreductase 1.5

1622 *msrA* methionine sulfoxide reductase A 1.5

179 putative oxidoreductase, flavodoxin -2.0

838 *gshR* glutathione reductase -1.5

**Transport**

857 *pyrP* uracil permease 1.6

1006 *ysaC* ABC transporter, ATP-binding protein 1.8

1007 *ysaB* ABC transporter, permease 1.5

995 *yclN* ferrichrome ABC transporter (permease) -1.5

1658 *nrgA* ammonium transporter NrgA -1.6

**Regulation**

112 transcriptional regulator, HTH RpiR family 1.7

856 *pyrR* pyrimidine regulatory protein PyrR 1.7

1397 *irvA* transcriptional repressor (HTH-XRE family) 2.1

1398* *irvR* repressor protein - phage associated

(HTH-XRE family) 1.6

1683 putative transcriptional regulator 1.6

1977 transcriptional regulator (HTH-XRE family) 1.6

1988* putative DNA-binding protein 2.7

136* putative transcriptional regulator, HTH-XRE

family protein -2.5

1657 *glnB*  nitrogen regulatory protein PII -1.7

**Housekeeping/structural**

20 *mreC* putative cell shape-determining protein MreC 1.8

393 TraB pilus assembly protein 1.5

865 *rpsP* 30S ribosomal protein S 16 1.8

1127 *rpsT* 30S ribosomal protein S 20 1.8

500 *yfiA* putative ribosome associated protein -1.5

609* *bsp* putative 40K cell wall protein precursor -3.6

1296 *yghU* glutathione S-transferase -1.5

1982 Type II secretory pathway -2.5

**Hypothetical/others**

391 hypothetical protein 3.2

423 hypothetical protein 1.6

501 hypothetical protein 1.7

752 putative metallopeptidase (SprT family) 1.6

866 RNA binding protein (HK domain) 1.8

925 hypothetical protein 1.6

1250 hypothetical protein 1.6

1395 hypothetical protein 8.7

1435 hypothetical protein 2.1

1436 hypothetical protein 2.9

1502 hypothetical protein 2.7

1774 hypothetical protein 1.8

1883 hypothetical protein 1.7

1907 hypothetical protein 1.6

1908 hypothetical protein 1.5

1925 hypothetical protein 2.1

1975 hypothetical protein 1.8

1976 hypothetical protein 1.6

2147 putative enzyme involved in cell wall degradation 1.6

285 hypothetical protein -1.5

503 hypothetical protein -1.8

545 hypothetical protein -1.6

673 hypothetical protein -1.7

941* hypothetical protein -1.7

1882* putative bacteriocin -2.5

1979 *ytxK* methyltransferase domain -1.6

1980 conserved hypothetical protein -2.7

________________________________________________________________________________________________________

Analysis was performed using Genespring GX 9 software.

^a^ SMU Numbers designate open reading frames based on *S. mutans* UA159 genome annotation.

^b^ genes that are potentially co transcribed are separated by a dash (-).

^c^ hyphen indicates unnamed gene

^d^ gene description based on nomenclature used at Oralgen database nomenclature (<http://www.oralgen.lanl.gov/>)

^e^ change in the transcript level of the *covR* mutant IBS10 strain compared to the wild type UA159 (∆*covR*/WT)

* putative promoter regions used for the EMSA analysis.
